# The Frequency and Determinants of Positive and Negative Officiating Interactions and the Relationships with Mental Ill-Health Symptoms in Sports Officials

**DOI:** 10.1007/s40279-025-02216-3

**Published:** 2025-04-10

**Authors:** Matthew McKeen, Clare Stevinson

**Affiliations:** School of Sport, Exercise and Health Sciences, Loughborough University, Loughborough, Leicestershire, LE11 3TU UK

## Abstract

**Background:**

Concerns exist about the prevalence of abuse directed towards sports officials and its impact on their mental health; however, sports officiating can also be a rewarding experience.

**Objectives:**

The present study aimed to identify the frequency and determinants of both positive (e.g. praise, appreciation, apologies) and negative (e.g. verbal abuse, physical abuse, social media abuse) officiating interactions and to examine their independent contributions to mental ill-health symptoms.

**Methods:**

An online, cross-sectional survey was distributed to current or former sports officials operating on a voluntary or paid basis in the United Kingdom (UK). Participants completed validated measures of mental ill-health symptoms (Depression, Anxiety, Stress Scale) and daily hassles (LIVES-Daily Hassles Scale) and reported the frequency with which they experienced positive and negative officiating interactions.

**Results:**

A total of 320 sports officials (73.8% male; mean age ± standard deviation (SD) = 49.99 ± 15.86 years) completed the survey with the most represented sports including cricket (30.3%), rugby union (24.4%) and football (soccer) [16.9%]. Regular positive officiating interactions were experienced by 90.0% of sports officials and regular negative officiating interactions by 22.2%. Logistic regression analyses revealed that male sports officials (odds ratio (OR) = 3.95; 95% confidence interval (CI) = 1.34, 11.65) were significantly more likely to experience regular positive interactions, while football officials (OR = 7.33; 95% CI = 1.38, 38.90) were significantly more likely to experience regular negative interactions. When controlling for daily hassles, age and sex, regular positive interactions were independently associated with lower odds of depression (OR = 0.38; 95% CI = 0.15, 0.92) and anxiety (OR = 0.33; 95% CI = 0.13, 0.82) symptoms. However, regular negative officiating interactions were not independently associated with mental ill-health symptoms.

**Conclusions:**

Positive officiating interactions were commonly experienced and independently predicted lower odds of mental ill-health symptoms. Approximately one fifth of officials reported regular negative interactions, although this was notably higher among football officials. No independent association existed between negative interactions and mental ill-health symptoms, and future research should determine if positive interactions help to offset the impact of negative ones for sports officials.

## Key Points

**Table:** 

An online, cross-sectional survey was used to identify the frequency and determinants of both positive and negative officiating interactions and to examine their independent contributions to mental ill-health symptoms in a sample of UK-based sports officials.
Positive interactions (e.g. praise, thanks and apologies) were regularly experienced by most officials (90%) and negative interactions (e.g. verbal, physical or online abuse) were regularly experienced by approximately one fifth (22%).
Logistic regression analyses revealed that male sports officials were significantly more likely to experience regular positive interactions, while football (soccer) officials were significantly more likely to experience regular negative interactions.
Whilst regular positive interactions were independently associated with lower odds of depression and anxiety symptoms, regular negative officiating interactions were not independently associated with mental ill-health symptoms.

## Introduction

There is an increasing focus within scientific literature [1, 2] as well as popular media [3, 4] on the abuse experienced by sports officials. Notably, several high-profile former officials of team sports have publicly discussed the abuse received during their officiating careers [5, 6]. This abuse has significant negative implications for the mental health of officials and contributes to the attrition crisis among officials prevalent across many sports [7, 8]. It is an important subject as officials are responsible for enforcing the rules of a given sport and are essential to ensuring that fixtures and competitions function effectively across all levels of participation [2]. Hence, there is a need to understand the nature of officiating interactions (both positive and negative) and their contributions to sports officials’ mental health [9, 10].

Research in sport psychiatry has predominantly focused on athlete populations, and there is growing evidence relating to the prevalence and determinants of mental disorders and their symptoms in elite participants [11–15]. Attention has extended to include coaches and other support staff [16–18] and, increasingly, the mental health of sports officials is being considered [19, 20]. The current study contributes to this field in assessing symptoms associated with common mental disorders among officials in relation to the positive and negative officiating interactions experienced.

### Negative Officiating Interactions and Mental Health

Verbal abuse (e.g. swearing, intimidation and threats) and physical abuse (e.g. pushing, punching and kicking) are two of the most prevalent officiating-related stressors for sports officials [2, 21]. However, recent research suggests some variation in the prevalence of abuse experienced across sports [1]. For instance, in the United Kingdom (UK), 93.8% of football (soccer) officials, 56.5% of cricket officials and 53.7% of rugby union officials have reported experiencing verbal abuse at some point during their officiating career, as well as 94.3% of Gaelic Games officials in Ireland [22–24]. Furthermore, 23.1% of Gaelic Games officials and 18.9% of football officials also reported experiencing physical abuse at some point during their officiating career [22, 24]. Together, these findings suggest that verbal and physical abuse are more commonly experienced among officials of some sports, particularly certain contact sports [1]. Whilst anger is positively correlated with physical and verbal aggression across all sport types, the relationship has been found to be strongest amongst contact sport competitors (e.g. martials arts, wrestling and football) [25]. In addition, contact sport athletes have been shown to possess greater on-field aggressiveness and to perceive aggressive behaviours during competition as more acceptable than non-contact sport athletes [26, 27].

To date, only one study has explored the relationship between experiences of abuse and mental ill-health symptoms among sports officials. Brick and colleagues demonstrated that greater feelings of distress resulting from physical and/or verbal abuse were associated with greater intentions to quit, lower mental wellbeing and more severe symptoms of both anxiety and depression in officials of Gaelic sports [24]. In addition, despite being experienced less frequently than verbal abuse, physical abuse was found to lead to greater distress [24]. The researchers suggested that the severity of abuse experienced might be a more important variable than its frequency and called for future research to incorporate measures of both frequency and severity [24]. It should also be noted that other sources of distress were not controlled for in the analysis.

### Positive Officiating Interactions and Mental Health

Whilst a general absence of appreciation for their efforts is a factor that contributes to the decision of some sports officials to discontinue [28, 29], sports officiating has also been shown to be a highly rewarding experience [30, 31]. For instance, sports officiating offers individuals an opportunity to maintain social relationships with former teammates, engage with the broader officiating community and serve the sport that they love [32]. Furthermore, despite the officiating-related stressors that sports officials are typically exposed to, officiating can contribute positively to the mental health of sports officials by providing them with a means to reduce and cope with the stress they experience away from sport [33].

Outside of a sports context, positive social interactions such as receiving praise and being thanked have been found to improve work engagement and facilitate both positive physical health and psychological wellbeing [34–36]. However, less is known about the prevalence of positive officiating interactions and their impact on the mental health of sports officials. Therefore, further research on this population is warranted [2].

### Purpose of the Present Study

Since research on abuse experienced by sports officials has often been examined in the context of a whole officiating career, it is unclear how regularly these negative interactions occur in some sports. Regular exposure to verbal or physical abuse might be expected to influence mental health more strongly than occasional past experiences. It is also unclear if experiencing positive interactions plays a part in protecting the mental health of officials. Current insight into the officiating characteristics that might make officials more vulnerable to abuse is limited [10]. Previous studies have suggested that younger or less experienced officials were more likely to experience verbal abuse [24, 37, 38], but other demographic or sport-related variables have not been examined in relation to frequency or type of officiating interactions. The nature of the sport (e.g. contact or non-contact) is also important to consider.

To address these issues and provide greater insight into the positive officiating interactions experienced in sport, the present study had two main aims. The first was to identify the frequency and determinants of both positive and negative officiating interactions within competitive sports. The second aim was to examine the independent contributions of regular positive and regular negative officiating interactions to the mental health of UK-based sports officials, having controlled for exposure to other sources of stress. On the basis of existing literature, it was hypothesised that negative officiating interactions would be more likely to be reported by officials of contact sports than non-contact sports (*H*_1_); regular negative officiating interactions would independently predict mental ill-health symptoms (*H*_2_); and regular positive officiating interactions would protect against mental ill-health symptoms (*H*_3_).

## Methods

### Research Design

Following institutional ethics approval (Ethics Review Sub-Committee of the School of Sport, Exercise and Health Sciences, Loughborough University, no. 13609), a cross-sectional survey was used to collect quantitative data during a 12-week period between April and July 2023 using the online survey platform Qualtrics (Provo, UT, USA). All participants provided informed consent before being presented with the survey questions. The survey was organised into three main sections: (1) experiences of positive and negative officiating interactions; (2) mental ill-health symptoms; and (3) demographic and officiating-related information.

### Participants

Inclusion criteria specified that all participants were over the age of 18 years, were able to understand English and were either a current or former sports official operating on a voluntary or paid basis in the UK. Recruitment involved contacting local sports clubs as well as regional and national officiating organisations across a range of sports via email and snowball sampling using the social media platforms Instagram, LinkedIn, Facebook and X (formerly Twitter).

### Measures

#### Experiences of Positive and Negative Interactions whilst Officiating

Participants were presented with 12 different interaction categories and asked to rate the frequency with which they had experienced each during their sports officiating career using six options (every fixture; every couple of fixtures; a couple of times a season; every few years; once or twice in my officiating career; never). These 12 interactions were classified as either positive (e.g. praise, thanks, apologies) or negative (e.g. physical abuse, verbal abuse, social media abuse) and as either moderate or strong. Owing to the subjectivity involved in judging the strength of an interaction, examples were provided for each category by drawing on recommendations made by Brick and colleagues [24]. Participant responses were not limited to game-day interactions and could include online interactions (Table 1).

**Table 1:** Officiating interaction categories and examples

Interaction categories	Examples
*Positive interactions*	
Moderate praise	Compliments on official’s performance
Moderate thanks	Thanks, handshakes
Moderate apology	In-person apology
Strong praise	Award or some other form of external recognition
Strong thanks	Written thank you
Strong apology	Written apology
*Negative interactions*	
Moderate verbal abuse	Swearing, name-calling, condescension
Moderate physical abuse	Pushing, shoving, slapping
Moderate social media abuse	Abusive or threatening messages, derogatory remarks in a post or comment
Strong verbal abuse	Threats of harm, death threats, racism
Strong physical abuse	Punching, kicking, head-butting, biting, hair-pulling
Strong social media abuse	Encouraging self-harming behaviours, racism

#### Mental Ill-Health Symptoms

The Depression, Anxiety and Stress Scale (DASS-21) is a 21-item self-report questionnaire comprising three 7-item sub-scales that assess depression, anxiety and stress symptoms [39]. Participants were asked to rate how strongly statements applied to them over the previous week using a 4-point Likert scale ranging from ‘0’ (did not apply to me at all) to ‘3’ (applied to me very much or most of the time). Scores are doubled, meaning that possible sub-scale totals range from 0 to 42, with higher scores indicating the presence of more severe mental ill-health symptoms. The DASS-21 has previously demonstrated acceptable test–retest reliability (ranging from *r* = 0.71 for depression to 0.81 for stress) and moderate-to-high concurrent validity with other measures of depression, anxiety and stress [40, 41]. Scores reaching thresholds of ≥ 10 (depression), ≥ 8 (anxiety) and ≥ 15 (stress) are interpreted as representing at least mild symptomology [39]. Cronbach’s alpha indicated good-to-excellent internal consistency in the present study (depression *α* = 0.90, anxiety *α* = 0.82 and stress *α* = 0.87).

#### Daily Hassles

To control for other sources of stress in the lives of participants, the 18-item LIVES Daily Hassle Scale (LIVES-DHS) was administered [42]. The scale assesses the cumulative experience of common minor disruptions to daily life on the basis of subjective appraisal of threat and coping and has shown independent associations with poorer life satisfaction, wellbeing and health. Participants respond to statements using a 4-point Likert scale ranging from ‘1’ (not at all) to ‘4’ (very much) with total scores ranging from 18 to 72. Higher scores indicate greater exposure to stress due to daily hassles. The LIVES-DHS has previously demonstrated excellent internal consistency (*α* = 0.95) and moderate concurrent validity with other measures of stress and health [42, 43]. In the present study, Cronbach’s alpha indicated acceptable internal consistency (*α* = 0.79).

#### Demographic and Officiating-Related Characteristics

Participants were asked to report demographic characteristics (sex, age and ethnicity) and officiating-related information (primary role, sports officiated, age-group and sex typically officiated, years of officiating experience, voluntary or paid role, and current or former official). Participants who had retired from officiating were asked one additional question relating to the extent to which the interactions that they had experienced during their sports officiating career had impacted their decision to stop officiating. This was measured on a 4-point Likert scale ranging from ‘1’ (a lot) to ‘4’ (not at all).

### Data Analysis

A total of 374 survey responses were exported into Microsoft Excel (version 2307). These data were screened, and all cases with less than 95% survey completion were deleted (*n* = 54). “Prefer not to say” responses were treated as missing data (< 2%). Consistent with existing literature suggesting that < 5% missing data are negligible, no imputations were made [44]. The final dataset of 320 responses was then analysed using IBM SPSS Statistics for Windows version 28.0 (IBM Corp, Armonk, NY, USA) with significance set at *p* < 0.05. Scale/sub-scale scores were calculated for the DASS-21 and LIVES-DHS, and internal consistency was assessed using Cronbach’s alpha.

Descriptive statistics (frequency, mean and standard deviation) were calculated for participant characteristics, officiating interactions and measured variables. Two thresholds were set for the frequency of officiating interactions. The first threshold was ‘experienced regularly’ (yes/no), which encompassed all interactions experienced at least every couple of fixtures. The second threshold was ‘experienced occasionally’ (yes/no), which encompassed all interactions experienced at least a couple of times a season. The 12 officiating interactions were then collapsed into two dichotomous variables: ‘regular positive interactions’ and ‘regular negative interactions’ for further analyses.

Chi-squared tests were used to examine characteristics of participants who experienced regular positive and regular negative officiating interactions. On the basis of guidance provided by MacMahon and Plessner, participants were classified as interactors (e.g. football referee, rugby union referee, cricket umpire), monitors (e.g. gymnastics judge, powerlifting judge, swimming judge) or reactors (e.g. football linesperson, tennis linesperson, track and field judge) using the information collected concerning sport(s) officiated and primary role [45]. Focusing specifically on the degree of interaction experienced by sports officials, monitors and reactors were then collapsed into a single category, which was compared against interactors in subsequent analyses. Sports officials were also dichotomised consistent with the classification of sports according to contact status reported elsewhere in the sports medicine literature [46]. Significant variables were included as covariates in logistic regression analyses to identify independent predictors of regular positive and regular negative officiating interactions.

The sample was then dichotomised on mental ill-health symptomology using the recommended cut-off values for the DASS-21 of ≥ 10 (depression), ≥ 8 (anxiety) and ≥ 15 (stress), representing at least mild symptomology [39]. Chi-squared tests were used to examine the univariate associations between regular officiating interactions (positive and negative) and mental ill-health symptoms (depression, anxiety and stress). Covariates were also examined using chi-squared tests (sex) or independent samples *t*-tests (age, daily hassles score). Logistic regressions were performed to examine the independent contribution of regular positive and regular negative officiating interactions to mental ill-health symptoms, controlling for participant age, sex and daily hassles score.

## Results

### Sample Characteristics

A total of 320 sports officials (73.8% male; mean age ± standard deviation (SD) = 49.99 ± 15.86 years) completed the survey. Most participants were active officials (94.7%) and were involved in officiating only one sport (90.6%) and on a voluntary basis (67.5%). Mean officiating experience (± SD) was 13.28 (± 11.55) years. Whilst officials from a total of 27 different sports completed the survey, the most represented sports included cricket (30.3%), rugby union (24.4%) and football (16.9%). The 27 sports were a mix of contact sports (American Football, football, rugby union, rugby league, and wheelchair rugby league) and non-contact sports (athletics, badminton, basketball, cricket, cross-country running, cycling, golf, gymnastics, hockey, motorsport, netball, powerlifting, rounders, rowing, swimming, table tennis, tennis, touch rugby, triathlon, volleyball, walking football, and wheelchair basketball). Most officials were categorised as interactors (71.3%).

### Officiating Interactions

#### Prevalence

The prevalence of positive and negative officiating interactions experienced on a regular and occasional basis are presented in Table 2. Moderate thanks was the most prevalent interaction type, with 86.3% of respondents receiving it regularly. Verbal abuse was the most prevalent negative interaction, received regularly by 20.9% of officials. When measured as two global constructs, some form of positive or some form of negative interaction was regularly experienced by 90.0% and 22.2% of officials, respectively. Of the 17 retired officials, the degree of impact of these interactions on decisions to stop officiating was reported as ‘a lot’ (*n* = 6), ‘somewhat’ (*n* = 2), ‘a little’ (*n* = 2) and ‘not at all’ (*n* = 7).

**Table 2:** Prevalence of positive and negative officiating interactions experienced on a regular and occasional basis (*n* = 320)

Interaction type	Regularly (at least every couple of fixtures)	Occasionally (at least a couple of times a season)
Moderate positive interactions	89.7%	96.9%
Moderate praise	55.6%	86.3%
Moderate thanks	86.3%	95.0%
Moderate apology	7.5%	44.1%
Strong positive interactions	11.6%	40.6%
Strong praise	7.8%	28.1%
Strong thanks	5.6%	24.1%
Strong apology	0.9%	3.1%
Moderate negative interactions	22.2%	48.4%
Moderate physical abuse	2.2%	4.1%
Moderate verbal abuse	20.9%	47.8%
Moderate social media abuse	0.9%	5.3%
Strong negative interactions	1.9%	7.2%
Strong physical abuse	0.6%	0.9%
Strong verbal abuse	1.6%	6.9%
Strong social media abuse	0.6%	1.3%
All positive interactions	90.0%	96.9%
All negative interactions	22.2%	48.4%

The differences in regular positive and regular negative officiating interactions based on officiating characteristics are presented in Table 3. Regular positive interactions were significantly more common among officials who were male (*χ*^2^ = 26.32; *p* < 0.001), interactors (*χ*^2^ = 21.03; *p* < 0.001) and who were overseeing contact sports (*χ*^2^ = 8.64; *p* = 0.003), adult competitors (*χ*^2^ = 8.27; *p* = 0.016) and male competitors (*χ*^2^ = 13.86; *p* < 0.001). Regular negative interactions were reported more by male officials (*χ*^2^ = 5.38; *p* = 0.020), interactors (*χ*^2^ = 16.92; *p* = 0.001), and those who were younger (*χ*^2^ = 15.65; *p* < 0.001), paid to officiate (*χ*^2^ = 47.23; *p* < 0.001), and overseeing contact sports (*χ*^2^ = 47.68; *p* < 0.001), female competitors (*χ*^2^ = 7.90; *p* = 0.019) and football (*χ*^2^ = 82.54; *p* < 0.001).

**Table 3:** Differences in regular positive and regular negative officiating interactions based on demographic and officiating-related characteristics (*n* = 320)

Officiating characteristics	Regular positive interactions	Regular negative interactions
*Sex*		
Female (*n* = 78)	75.6%	12.8%
Male (*n* = 236)	**95.3%**	**25.4%**
*Ethnicity*		
White (*n* = 296)	90.5%	21.6%
Non-white (*n* = 18)	77.8%	38.9%
*Age group*		
18–39 years (*n* = 82)	92.7%	**35.4%**
40–59 years (*n* = 140)	87.9%	22.9%
60 + years (*n* = 95)	90.5%	10.5%
*Sport officiated*^a^		
Football (*n* = 44)	88.6%	**72.7%**
Cricket (*n* = 77)	94.8%	10.4%
Rugby union (*n* = 70)	100.0%	20.0%
Other sports (*n* = 99)	**79.8%**	9.1%
*Official type*^b^		
Interactor (*n* = 228)	**94.3%**	**28.5%**
Monitor/reactor (*n* = 74)	74.7%	5.4%
*Contact/non-contact sport*		
Contact (*n* = 121)	**95.9%**	**42.1%**
Non-contact (*n* = 184)	85.3%	8.7%
*Age-group typically officiated*		
Children/youth (*n* = 31)	80.6%	25.8%
Adult/senior (*n* = 92)	**96.7%**	20.7%
Both (*n* = 197)	88.3%	22.3%
*Sex typically officiated*		
Female (*n* = 10)	60.0%	**50.0%**
Male (*n* = 120)	**95.0%**	26.7%
Both (*n* = 190)	88.4%	17.9%
*Officiating level*		
Local/community (*n* = 119)	92.4%	26.1%
District/county (*n* = 164)	88.4%	20.1%
National/international (*n* = 37)	89.2%	18.9%
*Length of officiating experience*		
0–5 years (*n* = 97)	85.7%	26.8%
6–15 years (*n* = 129)	89.9%	20.2%
16 + years (*n* = 94)	93.6%	20.2%
*Volunteer/paid official*		
Volunteer (*n* = 216)	90.7%	11.1%
Paid (*n* = 104)	88.5%	**45.2%**

Bold type indicates significantly different frequency for that sub-group based on chi-squared analyses

^a^Officials who officiated multiple sports were excluded (*n* = 30)

^b^Officials who possessed multiple roles or whose role was not indicated were excluded (*n* = 18)

#### Predicting Officiating Interactions

Sex, official type, contact/non-contact sport, age-group officiated and sex officiated were entered into the regression model for predicting regular positive officiating interactions (*χ*^2^ (7) = 38.16, *p* < 0.001), explaining 25.9% (Nagelkerke *R*^2^) of the variance (Table 4). However, only one variable reached significance as an independent predictor: male officials were more likely than female officials (odds ratio (OR) = 3.95; 95% confidence interval (CI) = 1.34, 11.65) to report regular positive interactions.

**Table 4:** Summary of logistic regression analyses for variables predicting regular positive and regular negative officiating interactions

Variable	*B*	SE	*χ*^2^	95% CI for OR			*P*-value
				OR	LL	UL	
*Regular positive officiating interactions*							
Sex (reference: female)							
Male	1.374	0.552	6.207	3.951	1.341	11.646	**0.013**
Official type (reference: interactor)							
Monitor/reactor	0.608	0.560	1.181	1.837	0.614	5.501	0.277
Contact/non-contact sport (reference: non-contact)							
Contact	1.268	0.698	3.297	3.552	0.904	13.955	0.069
Age-group officiated (reference: both adult and children)							
Children/youth only	− 0.336	0.598	0.315	0.715	0.221	2.310	0.575
Adult/senior only	1.266	0.837	2.289	3.548	0.688	18.299	0.130
Sex officiated (reference: both male and female)							
Female only	− 0.483	0.830	0.338	0.617	0.121	3.139	0.561
Male only	− 0.401	0.690	0.338	0.669	0.173	2.591	0.561
Constant	0.685	0.358	3.669	1.984			0.055
*χ*^2^ (7) = 38.162							**< 0.001**
*Regular negative officiating interactions*							
Sex (reference: female)							
Male	− 0.336	0.625	0.289	0.715	0.210	2.433	0.591
Age-group (reference: 60 + years)							
40–59 years	0.181	0.517	0.122	1.198	0.435	3.298	0.726
18–39 years	0.461	0.568	0.658	1.586	0.521	4.830	0.417
Official type (reference: monitor/reactor)							
Interactor	1.045	0.810	1.663	2.843	0.581	13.915	0.197
Contact/non-contact sport (reference: non-contact)							
Contact	1.455	1.052	1.914	4.286	0.545	33.690	0.167
Sex officiated (reference: both male and female)							
Female only	1.780	0.968	3.380	5.932	0.889	39.590	0.066
Male only	0.522	0.449	1.350	1.685	0.699	4.061	0.245
Officiating employment status (reference: volunteer)							
Paid	0.290	0.528	0.301	1.336	0.474	3.763	0.584
Sport (reference: all other sports)							
Football	1.992	0.852	5.470	7.329	1.381	38.902	**0.019**
Cricket	0.122	0.824	0.022	1.129	0.225	5.679	0.883
Rugby union	− 0.630	0.953	0.437	0.533	0.082	3.449	0.509
Constant	− 2.458	0.879	7.823	0.086			0.005
*χ*^2^ (11) = 89.037							**< 0.001**

Bold type indicates significance at the *p* < 0.05 level (two-tailed)

*B* unstandardised beta coefficient, *SE* standard error, *χ*^2^ Wald test statistic, *OR* odds ratio, *CI* confidence interval, *LL* lower limit, *UL* upper limit

For predicting negative officiating interactions, sex, age, official type, contact/non-contact sport, sex officiated, paid/voluntary status and sport were entered into the regression model (*χ*^2^ (11) = 89.04, *p* < 0.001), explaining 43.9% (Nagelkerke *R*^2^) of the variance (Table 4). Again, only one variable reached significance as an independent predictor: football officials (OR = 7.33; 95% CI = 1.38, 38.90) were more likely to report regular negative interactions.

### Officiating Interactions and Mental Ill-Health Symptoms

#### Prevalence of Mental Ill-Health Symptoms

Overall, 31.6% (*n* = 101), 24.4% (*n* = 78) and 27.2% (*n* = 87) of the sample reported experiencing at least mild severity depression, anxiety and stress symptoms, respectively. The differences in regular positive and negative interactions between officials with and without mental ill-health symptoms are presented in Table 5.

**Table 5:** Differences in regular positive and regular negative interactions between officials with and without mental ill-health symptoms

	Depression		Anxiety		Stress	
	No (*n* = 219)	Yes (*n* = 101)	No (*n* = 242)	Yes (*n* = 78)	No (*n* = 233)	Yes (*n* = 87)
Positive interactions	**93.1%**	83.2%	**92.5%**	82.1%	**92.3%**	83.9%
Negative interactions	18.3%	**30.7%**	19.1%	32.1%	21.9%	23.0%
*Sex*						
Male	71.6%	28.4%	78.0%	22.0%	78.8%	21.2%
Female	60.3%	39.7%	70.5%	29.5%	56.4%	**43.6%**
Age (mean ± SD)	**53.12** ± 15.03	43.02 ± 15.36	**52.98** ± 14.77	40.42 ± 15.30	**53.63** ± 14.56	40.06 ± 15.03
Daily hassles score (mean ± SD)	23.95 ± 4.93	**29.17** ± 7.17	24.35 ± 5.07	**29.47** ± 7.83	24.06 ± 4.67	**29.79** ± 7.82

Bold type indicates significantly different frequencies based on chi-squared analyses (regular positive and regular negative officiating interactions, sex) or means based on independent samples* t*-tests (age and daily hassles score)

Participants who met the threshold for depression symptoms were significantly more likely to be younger (*t*_(314)_ = 5.51, *p* < 0.001), to report a higher daily hassles score (*t*_(317)_ = 7.57, *p* = 0.006), to experience regular negative officiating interactions (*χ*^2^ = 6.08; *p* = 0.014) and less likely to experience regular positive officiating interactions (*χ*^2^ =  − 7.57; *p* = 0.006). Sex was not associated with depression symptoms (*χ*^2^ = 3.52; *p* = 0.061).

Participants who met the threshold for anxiety symptoms were significantly more likely to be younger (*t*_(314)_ = 6.44, *p* < 0.001), to report a higher daily hassles score (*t*_(317)_ = 6.76, *p* < 0.001), to experience regular negative officiating interactions (*χ*^2^ = 5.72; *p* = 0.017) and less likely to experience regular positive officiating interactions (*χ*^2^ =  − 7.17; *p* = 0.007). Sex was not associated with anxiety symptoms (*χ*^2^ = 1.79; *p* = 0.181).

Participants who met the threshold for stress symptoms were significantly more likely to be female (*χ*^2^ = 15.02; *p* < 0.001), younger (*t*_(315)_ = 7.29, *p* < 0.001), to report a higher daily hassles score (*t*_(318)_ = 7.89, *p* < 0.001) and less likely to experience regular positive officiating interactions (*χ*^2^ =  − 4.93; *p* = 0.026). Regular negative officiating interactions were not associated with stress symptoms (*χ*^2^ = 0.04; *p* = 0.833).

#### Predicting Mental Ill-Health Symptoms

Three logistic regression analyses were performed to identify the independent contributions of regular positive and negative officiating interactions to depression, anxiety and stress symptoms, with age, sex and daily hassle score included as covariates (Table 6).

**Table 6:** Summary of logistic regression analyses for variables predicting at least mild severity mental ill-health symptoms

Variable	*B*	SE	*χ*^2^	95% CI for OR			*P*-value
				OR	LL	UL	
*Depression*							
Regular positive interactions (reference: no)							
Yes	− 0.981	0.456	4.631	0.375	0.154	0.916	**0.031**
Regular negative interactions (reference: no)							
Yes	0.195	0.334	0.342	1.216	0.632	2.340	0.559
Sex (reference: female)							
Male	− 0.004	0.334	0	0.996	0.517	1.917	0.990
Age	− 0.035	0.009	14.461	0.966	0.948	0.983	**< 0.001**
Daily hassles score	0.131	0.026	25.050	1.139	1.083	1.199	**< 0.001**
Constant	− 1.657	0.951	3.036	0.191			0.081
*χ*^2^ (5) = 67.152							**< 0.001**
*Anxiety*							
Regular positive interactions (reference: no)							
Yes	− 1.122	0.471	5.681	0.326	0.129	0.819	**0.017**
Regular negative interactions (reference: no)							
Yes	0.123	0.355	0.120	1.131	0.564	2.270	0.729
Sex (reference: female)							
Male	0.189	0.360	0.275	1.208	0.597	2.445	0.600
Age	− 0.048	0.010	22.499	0.953	0.934	0.972	**< 0.001**
Daily hassles score	0.108	0.026	17.884	1.114	1.060	1.171	**< 0.001**
Constant	− 0.912	0.977	0.872	0.402			0.350
*χ*^2^ (5) = 63.864							**< 0.001**
*Stress*							
Regular positive interactions (reference: no)							
Yes	− 0.541	0.486	1.237	0.582	0.225	1.510	0.266
Regular negative interactions (reference: no)							
Yes	− 0.761	0.399	3.633	0.467	0.214	1.022	0.057
Sex (reference: female)							
Male	− 0.495	0.345	2.066	0.609	0.310	1.197	0.151
Age	− 0.057	0.011	29.374	0.945	0.925	0.964	**< 0.001**
Daily hassles score	0.141	0.028	26.140	1.152	1.091	1.216	**< 0.001**
Constant	− 1.059	1.008	1.104	0.347			0.293
*χ*^2^ (5) = 87.889							**< 0.001**

Bold type indicates significance at the *p* < 0.05 level (two-tailed)

*B* unstandardised beta coefficient, *SE* standard error, *χ*^2^ Wald test statistic, *OR* odds ratio, *CI* confidence interval, *LL* lower limit, *UL* upper limit

For depression, there was a significant model (*χ*^2^ (5) = 67.15, *p* < 0.001), explaining 27.2% (Nagelkerke *R*^2^) of the variance with three independent predictors: regular positive interactions, age and daily hassles score. The odds of experiencing depression symptoms decreased by 0.97 (95% CI = 0.95, 0.98) for every unit increase in age and by 0.38 (95% CI = 0.15, 0.92) when regular positive interactions were experienced but increased by 1.14 (95% CI = 1.08, 1.20) for every unit increase in daily hassles score.

Similarly, the model for anxiety was significant (*χ*^2^ (5) = 63.86, *p* < 0.001), explaining 27.7% (Nagelkerke *R*^2^) of the variance with the same three independent predictors: regular positive interactions, age and daily hassle score. The odds of experiencing anxiety symptoms decreased by 0.95 (95% CI = 0.93, 0.97) for every unit increase in age and by 0.33 (95% CI = 0.13, 0.82) when regular positive interactions were experienced but increased by 1.11 (95% CI = 1.06, 1.17) for every unit increase in daily hassles score.

A significant model was also produced for stress (*χ*^2^ (5) = 87.89, *p* < 0.001), explaining 35.8% (Nagelkerke *R*^2^) of the variance with only age and daily hassles score as independent predictors. The odds of experiencing stress symptoms decreased by 0.95 (95% CI = 0.93, 0.96) for every unit increase in age but increased by 1.15 (95% CI = 1.09, 1.22) for every unit increase in daily hassles score.

## Discussion

To further understanding of the abuse of sports officials, it is also important to consider the positive interactions experienced through sports officiating. This study identified the frequency and determinants of positive and negative interactions reported by UK sports officials and explored associations with mental ill-health symptoms. The results indicated that positive interactions, particularly receiving thanks and praise, were very common (90% experienced them on a regular basis). Negative interactions, usually verbal abuse, were also quite common (22% reported experiencing regularly). Regular positive interactions were independently associated with being male, while overseeing football players was the only independent predictor of regular negative interactions. Finally, regular positive interactions were associated with lower odds of depression and anxiety symptoms, but no relationships with mental ill-health symptoms existed for regular negative interactions.

### Positive Officiating Interactions

Positive interactions were experienced regularly by a high proportion of participants, with only the official’s sex being a significant independent predictor. The finding that male officials were more likely to encounter praise, thanks and apologies than their female counterparts is compatible with the reported gender inequality within some sporting contexts. Difficulty in gaining acceptance and respect has been discussed by female officials in several sports, including football [47, 48], rugby union [49] and basketball [29]. This challenge for women extends to other domains of sports leadership, such as coaching, and a combination of individual, organisational and societal factors have been implicated [50]. Sociocultural norms were shown to be particularly influential on officiating experiences in a recent study of 3214 female officials involved in 69 sports across 40 European countries. Higher levels of gender equality within society were positively associated with intentions to continue in officiating roles [51].

The prevalence of positive interactions was significantly higher among officials classed as interactors (94%) than in the monitors/reactors category (75%) and higher among those officiating contact sports (96%) than non-contact sports (85%). However, neither variable was a significant independent predictor of positive interactions in the subsequent regression analysis, suggesting that the frequency of positive interactions is more likely sport specific. For example, in the current study, reports of regular positive interactions were notably high for officials of the team sports of rugby union (100%), cricket (95%) and football (89%). This might be, at least in part, a reflection of each sport’s founding principles and recent attempts to promote positive interactions on and off the field. For instance, World Rugby includes respect for team-mates, opponents and match officials as one of the core values, alongside integrity, discipline, solidarity and passion [52]. Similarly, Marylebone Cricket Club, the guardian of the Laws of Cricket, cites respect towards captains, teammates, opponents and the authority of the umpires as central to the ‘Spirit of Cricket’ [53]. In football, numerous campaigns have been launched to improve behaviour on and off the pitch, such as the Football Association’s (FA) ‘Respect’ programme, which was introduced in 2008 and refreshed in 2018 with the additional strapline “we only do positive” [54].

### Negative Officiating Interactions

Regular negative interactions were reported by approximately one fifth of the overall sample of officials. This was markedly higher among football officials, with 73% indicating that they experienced negative officiating interactions regularly compared with only 20% of rugby union officials and 10% of cricket officials. This figure is slightly higher than the 60% of English football referees who have previously reported experiencing verbal abuse at least every couple of matches, with a further 19% indicating that they had experienced physical abuse at some point during the season [22]. Officiating footballers was the only independent predictor of regular negative interactions. Ongoing concerns about the rise in verbal and physical abuse of officials in football have been expressed, and numerous attempts to address this have been made [55]. For instance, in the build-up to the European Championships in the summer of 2024, the Union of European Football Associations (UEFA) asked that all teams ensured their captain was the only player who spoke to the referee during the match, warning that any player who failed to adhere to this new rule would be awarded a yellow card [56]. Following its perceived success, UEFA announced their intention to extend this policy to all European club competitions from the start of the 2024/2025 season [57]. Another recent measure trialled by the FA in England involves providing grassroots officials with bodycam technology [3]. Although it is too early to definitively determine the extent to which this intervention has improved officiating experiences, early reports are promising, and the trial has been extended [58].

As hypothesised, regular negative interactions were reported significantly more by officials of contact sports (42%) than non-contact sports (9%). However, this variable did not reach significance as an independent predictor of negative interactions in the subsequent regression analysis. Similarly, despite significant differences between interactors (29%) and monitors or reactors (5%), the official type variable was not a significant independent predictor. Since the same pattern of findings was observed for these variables in relation to positive interactions, it supports the suggestion that interactions with officials depend more on individual sport characteristics than broader categories [1]. Rugby union and football, for example, are both contact sports in which referees are highly involved in an interactor role. However, the frequency of negative interactions was very different between the two sports (20% versus 72.2%, respectively) and may be attributable to the culture within each specific sport. Among supporters, greater acceptance of verbal abuse towards referees has been shown for football compared with rugby union [59].

The prevalence of negative interactions was significantly higher among sports officials aged 18–39 years (35%) compared with older participants. This finding is consistent with recent research involving Gaelic Games officials, which found that younger and less experienced sports officials reported more frequent verbal abuse [24]. Younger sports officials are also considered less likely to possess the necessary skills to manage interpersonal conflict and/or cope with negative interactions effectively [1, 22, 38]. Interestingly, neither sports official’s age nor level of experience were found to independently predict regular negative officiating interactions in the subsequent regression analysis. While this may indicate the importance of controlling for additional variables, it is also worth noting that participants in the current study were all adults, whereas other studies have included officials younger than 18 years [38, 60, 61].

### Officiating Interactions and Mental Ill-Health Symptoms

The high prevalence of regular positive interactions reported among officials adds weight to previous research indicating officiating to be a rewarding experience [30, 31]. For instance, a study of Australian Rules football umpires revealed that positive social interactions drove their enjoyment and commitment and outweighed negative elements of officiating [62]. Previous research has demonstrated that both quantity and quality of momentary social interactions are associated with wellbeing [36], and that diversity of interactions with known and unknown individuals is the strongest contributor to wellbeing [63]. This evidence is compatible with the findings in the current study that regular positive interactions were independently associated with lower odds of depression and anxiety symptoms. These results support earlier suggestions that elements of officiating can contribute to protecting mental health [33, 62].

Two thirds of the current sample were unpaid volunteers. Research among volunteers in sport [64] and other domains [65, 66] has indicated the value of volunteer activities for personal wellbeing. Definitions of mental health often emphasise self-actualisation, resilience, productivity and making a contribution, alongside the absence of mental illness [67]. Engaging in prosocial activities can be rewarding for intrinsic reasons or indirectly through skill development or social interaction mechanisms [68]. Volunteering within sport in various capacities (e.g. coach, official, administrative, event operations) demonstrated a causal relationship with subjective wellbeing in an analysis involving nearly 53,000 sport volunteers from 28 European countries [69].

Contrary to the study hypothesis, regular negative interactions were not independently associated with mental ill-health symptoms after controlling for age, sex and daily hassles score. The prevalence of mental ill-health symptoms in the current sample of officials was relatively high (32% depression, 27% stress and 24% anxiety). In comparison, the figures reported from a large sample of adults broadly representative of the UK population using the same measurement tool were 18% for both depression and stress and 11% for anxiety [70]. Given that these data were collected 20 years earlier, some of the difference may reflect the national trends in common mental health problems observed over this period [71]. The difference may also be partly due to the non-probability sampling method of the study. Research shows, for instance, that individuals with a mental health problem are more likely to respond to advertisements for studies investigating mental health than the remainder of the population [72]. Nonetheless, the prevalence figures are compatible with other recent studies of mental health among sports officials which have reported prevalence ranges of 5–45% for depression, anxiety and stress using various validated instruments [24, 73–77]. Collectively, these findings suggest that the mental health of officials warrants greater attention, alongside the consideration given to other groups in the competitive sport domain such as current and former athletes [11–15] and coaching staff [16–18].

### The Role of Effective Officiating Communication

The role of communication and other interpersonal skills is increasingly discussed in relation to successful performance and coping with the pressures of officiating [78, 79]. Whilst the present study focused primarily on interactions received by sports officials, existing research highlights the two-way nature of communication as critical to effective officiating [80, 81]. Cunningham and colleagues [82] identified four key themes of effective officiating communication: personal qualities of the official (e.g. respectful, professional, empathic, calm, resilient); one-way communication (e.g. direction-giving and impression management); situation monitoring (e.g. interpreting players’ emotional responses in the context of the game); and skilled interaction (adapting communication appropriately for the specific situation). Notably, situation monitoring and skilled interaction are suggested to be particularly important for resolving interpersonal conflict when officiating [82], potentially reducing the negative interactions officials receive during and after games.

The relevance of these communication skills may vary across officiating roles. For example, situational monitoring and skilled interaction may be more critical aspects of communication for interactors, who are more actively involved in game management and experience a higher volume and complexity of interactions compared with monitors or reactors [45]. Nonetheless, monitors and reactors, who rely more heavily on observation and reactive communication [45], may still benefit from interpersonal skills to manage occasional high-pressure interactions. Tailoring training approaches to these role-specific demands could further enhance officiating effectiveness.

### Applied Implications

In light of the findings from the present study, several suggestions can be offered to help sporting organisations promote and protect the mental health of sports officials whilst also addressing the high attrition rates prevalent across many sports.

Within existing literature there have been several calls for sporting organisations to develop preventative policies that adopt a zero-tolerance approach to reduce the abuse directed towards sports officials [8, 19]. Whilst such policies are vital, organisations responsible for employing sports officials may also benefit from initiatives that promote the positive side of officiating. The results of the present study indicated that positive interactions, particularly receiving thanks and praise, were very common (90% experienced them on a regular basis) and, importantly, were associated with lower odds of depression and anxiety symptoms. Highlighting these positive experiences could not only improve the public perception of the role but also attract and retain individuals who may otherwise be deterred by the challenges of officiating.

The association between regular positive interactions and lower odds of depression and anxiety symptoms identified in this study underscores the importance of establishing organisational cultures that celebrate, recognise and appreciate sports officials. Employee recognition is widely recognised as an indicator of employee motivation, satisfaction and performance [83] as well as a means by which organisations can improve employee retention [84, 85]. However, a perceived lack of appreciation and organisational support has consistently been identified as a contributing factor in sports officials’ decisions to discontinue elsewhere in literature [1, 28, 29, 60, 86, 87]. To address this, sporting organisations should ensure that officials’ contributions are regularly acknowledged through both informal and formal recognition systems such as award ceremonies or public expressions of gratitude. Similar calls have been made within a higher education context in recent years [88].

In addition to monitoring the mental health of officials and ensuring the availability of psychological support, the value of mentoring programmes as a retention strategy has been highlighted in recent research, particularly for younger officials. For instance, the knowledge and experience of established sports officials can help less experienced individuals develop effective coping strategies for the pressures of the role [2, 22, 31]. Younger sports officials have also noted the positive impact that senior officials can have on their development [38]. The inclusion of formal mentoring schemes may help address the perceived lack of organisational support already highlighted [1, 60, 86, 87]. Similarly, the presence of role models has been found to exert a positive trickle-down effect on the retention of already active referees and the recruitment of new referees operating in German football [89].

Finally, a recent systematic appraisal of training and development approaches for sports officials indicated some value of psychological skills training (PST) with a positive impact on officiating performance [90]. Whilst further research is needed to explore how PST can be refined to develop the necessary communication and interpersonal skills to manage positive and negative interactions as an official [90], its inclusion within training and development programmes could better equip sports officials for the demands of the role.

### Study Limitations and Future Research Directions

This study provides crucial information on the positive interactions experienced by sports officials, which have rarely been included in previous research on abuse of referees and umpires. The associations demonstrated between regular positive interactions and lower odds of mental ill-health symptoms indicate the merit in further investigation of the potential benefits of officiating. Nonetheless, it is important to consider several limitations of the current study and provide suggestions for advancing and improving future research in this area.

Although the sample included officials from a wide range of sport types, the numbers were insufficient in most cases to provide results for specific sports. Therefore, the reported findings for the whole sample may be masking unique profiles of officiating interactions or differing relationships with mental health for some sports. The available data indicated some clear differences between some sports, such as the higher prevalence of negative interactions among football officials than those from other sports. Hence, greater proportions of certain sports within a sample have potential to influence overall findings. Future research on more homogenous officiating groups, as well as broad samples, can add to the growing understanding of the importance and impacts of both positive and negative interactions.

Moreover, the relatively small proportion of paid officials (32%), female officials (24%) and those of a non-white ethnic origin (6%) also restricted analyses based on these characteristics, and future research should aim for greater demographic diversity. Further insight on the status of paid officials (e.g. full-time, part-time, per game) would enable comparisons based on professional and amateur officials and different qualification levels. Marital status is another participant characteristic that merits inclusion in studies to strengthen understanding of the determinants of officiating experiences and associated consequences. Notably, sports officials who were married or in a committed relationship have previously reported less severe mental ill-health symptoms and higher wellbeing than officials who were single, divorced, separated or widowed [73].

The majority of the study participants (95%) were active officials. As a result, survival bias might have influenced the reporting of mental ill-health symptoms and officiating interactions, with some sports officials who experienced mental ill-health and/or negative officiating interactions likely to have left the sport [8]. Over half of the 17 retired officials in the current study indicated that negative interactions had some influence on the decision to stop officiating. Further research on retired officials would help clarify the role of abuse in the attrition of sports officials.

While a large volume of data was collected for this study, the sources of positive and negative officiating interactions were not recorded. Existing research suggests officials can experience negative interactions from a range of sources, including players, coaches, club officials and spectators [1, 23, 24, 60, 91], with spectators frequently identified as the most frequent source of negative interactions [92, 93]. Future research to determine how sources may vary depending on the sporting context would be informative, along with the extent to which the impact of interactions on sports officials’ mental health may differ depending on its source.

Analyses were based on officiating interactions defined as two global constructs (regular positive interactions and regular negative interactions), each composed of three officiating interaction types measured at both a moderate and strong intensity. Consequently, insight into any differences between specific interactions was lost, and future research that assesses officiating interactions without aggregating in this way could expand current understanding. We echo Brick and colleagues' call for future research to incorporate measures of both frequency and severity [24].

In addition, this study did not specifically consider other personal resources that have been shown to protect against mental ill-health symptoms. According to Lazarus and Folkman’s transactional model, the way in which individuals appraise and respond to stressors depends on the degree of perceived threat and coping ability [94]. An individual’s coping resources play a crucial role in mitigating the negative effects of threatening experiences by influencing these appraisal and response processes [95]. In addition, adequate social support networks can help to increase confidence, reduce mental health stigma and develop more effective coping strategies to deal with stress [96, 97]. Furthermore, sports officials with high levels of self-efficacy report greater commitment to the role, fewer negative officiating interactions and lower levels of stress [98, 99]. Future research that includes measures of protective resources such as social support, coping resources and self-efficacy would help to develop a more nuanced understanding of the relationship between officiating interactions and sports officials’ mental health.

Finally, the cross-sectional design further limits the conclusions that can be drawn about the relationships between officiating interactions and mental health, since causation cannot be established. Future prospective research would enable the rate and causes of attrition among officials to be captured along with a clearer examination of the changes in mental health in association with positive and negative interactions experienced over time.

## Conclusions

This research has contributed to literature on sports officials by focusing directly on the positive officiating interactions encountered alongside negative experiences, which have generally not been considered in literature. In doing so, it has enabled the independent relationship between officiating interactions and mental ill-health symptoms to be examined, controlling for external sources of stress by measuring daily hassles, as well as age and sex. In the current study, regular positive interactions were commonly experienced and were independently associated with lower odds of mental-ill health symptoms. In contrast, negative officiating interactions were experienced regularly by approximately one fifth of sports officials but were not associated with mental ill-health symptoms after controlling for other variables. Future research with more diverse samples and including retired officials would help strengthen understanding of demographic and sporting characteristics most likely to encounter positive and negative officiating interactions.

## Declarations

### Data availability

To protect study participant privacy, data are not publicly available but can be accessed via Loughborough University repository upon reasonable request.
